# ARG098, a novel anti-human Fas antibody, suppresses synovial hyperplasia and prevents cartilage destruction in a severe combined immunodeficient-HuRAg mouse model

**DOI:** 10.1186/1471-2474-11-221

**Published:** 2010-09-27

**Authors:** Noriko Odani-Kawabata, Miwa Takai-Imamura, Osamu Katsuta, Hiroshi Nakamura, Kusuki Nishioka, Keiko Funahashi, Tsukasa Matsubara, Minoru Sasano, Hiroyuki Aono

**Affiliations:** 1 Research & Development Center, Santen Pharmaceutical Co., Ltd., 8916-16, Takayama-cho, Ikoma-shi, Nara, Japan; 2 Institute of Medical Science, St. Marianna University School of Medicine, 2-16-1, Sugo, Miyamae-ku, Kawasaki-shi, Kanagawa, Japan; 3 Matsubara Mayflower Hospital, 944-25, Fujita, Katou-shi, Hyogo, Japan

## Abstract

**Background:**

The anti-human Fas/APO-1/CD95 (Fas) mouse/human chimeric monoclonal IgM antibody ARG098 (ARG098) targets the human Fas molecule. The cytotoxic effects of ARG098 on cells isolated from RA patients, on normal cells *in vitro*, and on RA synovial tissue and cartilage *in vivo *using implanted rheumatoid tissues in an SCID mouse model (SCID-HuRAg) were investigated to examine the potential of ARG098 as a therapy for RA.

**Methods:**

ARG098 binding to each cell was analyzed by cytometry. The effects of ARG098 on several cells were assessed by a cell viability assay *in vitro*. Effects on the RA synovium, lymphocytes, and cartilage were assessed *in vivo *using the SCID-HuRAg mouse model.

**Results:**

ARG098 bound to cell surface Fas molecules, and induced apoptosis in Fas-expressing RA synoviocytes and infiltrating lymphocytes in the RA synovium in a dose-dependent manner. However, ARG098 did not affect the cell viability of peripheral blood mononuclear cells of RA patients or normal chondrocytes. ARG098 also induced apoptosis in RA synoviocytes and infiltrating lymphocytes in the RA synovium *in vivo*. The destruction of cartilage due to synovial invasion was inhibited by ARG098 injection in the modified SCID-HuRAg mouse model.

**Conclusions:**

ARG098 treatment suppressed RA synovial hyperplasia through the induction of apoptosis and prevented cartilage destruction *in vivo*. These results suggest that ARG098 might become a new therapy for RA.

## Background

Rheumatoid arthritis (RA) is a chronic, inflammatory, and proliferative autoimmune disease characterized by synovial pannus formation that causes joint pain and swelling [1]. Inflammation and proliferation of synoviocytes erodes the cartilage and leads to joint bone destruction. One of the most important pathogenetic factors in RA that worsens the disease is synovial hypertrophy. Consequently, an effective strategy for RA treatment is the removal of synovial hyperplasia to prevent cartilage destruction and increase the quality of life (QOL) of patients [1,2].

Apoptosis is an essential biological system for development, differentiation, and homeostasis [3]. Apoptotic cell death exists in the RA synovium [4] but cell proliferation dominates in RA-affected joints, indicating that the balance between cell growth and death in the synovium collapses in RA joints [5] or that some Fas-resistant signals are activated in the RA synovium [6,7].

Most of the mechanisms affecting the abnormal overgrowth in the RA synovium remain unclear but a large section of the RA synovium is sensitive to apoptosis signals, and the anti-Fas/APO-1/CD95 (Fas) antibody induces apoptosis in the RA synovium [4] and decreases joint swelling [8]. On this basis, we hypothesized that this anti-Fas antibody would restore balance and reduce hyperplasia in RA joints. Inducing apoptosis in the RA synovium is effective for the suppression of arthritis [5,9,10].

On the other hand, because the anti-Fas antibody is a potent inducer of apoptosis, it is possible that the induction of apoptosis in non-target cells or organs could lead to severe adverse effects. For example, functional APO-1/Fas molecules are expressed on the surface of human hepatocytes [11] and induction of apoptosis in murine hepatocytes by the anti-Fas antibody has been shown to be lethal [12].

In this study, we evaluated the efficacy of a novel anti-human Fas mouse/human chimeric monoclonal antibody, ARG098, and its toxicity towards non-target cells or organs. In addition, the potency of ARG098 has been assessed *in vivo *using severe combined immunodeficient (SCID) mice implanted with the RA synovium (SCID-HuRAg) [13]. This murine model mimics human RA-affected joints [14-17].

## Methods

### Reagents

ARG098 was constructed by ligating the variable region of an anti-human Fas/APO-1/CD95 mouse monoclonal antibody, anti-APO-1 [18], with the constant region of the human antibody. The plasmid was transfected into the ARG098 mouse myeloma cell line, and the ARG098 antibody was secreted in the culture medium and purified.

The sources of the other materials used in this study are as follows: human IgM was obtained from ICN Biomedicals Inc. (Aliso Viejo, CA, USA), chondrocyte basal medium supplemented with chondrocyte growth supplement was obtained from Cell Applications, Inc. (San Diego, CA, USA), and the neutralizing antibody anti-human APO-1/Fas (SM1/23) was obtained from Bender Medsystems GmbH (Vienna, Austria). Recombinant human tumor necrosis factor-α (TNF-α) and recombinant human interleukin-1β (IL-1β) were purchased from R & D Systems (Minneapolis, MN, USA). The Cell Counting Kit-8 was obtained from Dojindo Laboratories (Kumamoto, Japan), and the CellTiter-Glo™ Luminescent Cell Viability Assay and CytoTox96^® ^Non-Radioactive Cytotoxicity Assay Kits were purchased from Promega (Madison, WI, USA). The Annexin V/FITC Kit was obtained from Takara Bio Inc. (Shiga, Japan), and the PerCP-labeled anti-human CD4 antibody, PE-labeled anti-human CD8 antibody, PE-labeled mouse IgG_1_, and PerCP-labeled mouse IgG_1 _were purchased from BD Biosciences (Franklin Lakes, NJ, USA). *In situ *apoptosis detection kit was purchased from Takara Bio Co., Ltd. (Shiga, Japan).

### Cell isolation and cell culture (cells and tissues)

The experimental procedure followed the Declaration of Helsinki, and was approved and monitored by the Ethics Review Board on Human Tissue Research of Santen Pharmaceutical Co., Ltd., St. Marianna University School of Medicine and Matsubara Mayflower Hospital. All synoviums, cartilages and peripheral blood samples were taken from RA patients with their informed consent. RA synoviums and cartilage samples were supplied immediately after synovectomy for RA treatment. None of the RA patients were treated with biological agents.

Synoviums were minced with scissors and digested in DMEM containing 1 mg/mL collagenase at 37°C for 30 min. Isolated cells were suspended in Ham's F-12 medium supplemented with 10% FBS, 100 U/mL penicillin, and 100 μg/mL streptomycin, and cultured at 37°C in 5% CO_2_. After 1 day of incubation, the cells which did not adhere (RA synovium-infiltrating lymphocytes) were separated from those that adhered to the culture plates (RA synoviocytes). RA synoviocytes prepared using the same methods were heteromorphous and belonged to three different types: dendritic cells, macrophage cells, and fibroblast-like cells [4], however, the majority of our tested cell population may be synovial fibroblasts. All experiments were performed using RA synoviocytes within the fifth passage, and using RA synovium-infiltrating lymphocytes without further passage.

Peripheral blood mononuclear cells (PBMCs) were isolated from the peripheral blood of RA patients (the same patients who underwent synovectomy) by density centrifugation. All experiments with RA synoviocytes, RA synovium-infiltrating lymphocytes, and PBMCs were performed using 5 different samples derived from 5 RA patients.

### Human chondrocyte culture

Normal human chondrocytes were purchased from Cell Applications, Inc., and were three-dimensionally cultured in alginate beads in chondrocyte basal medium supplemented with chondrocyte growth supplement and preserved at 37°C in 5% CO_2 _to maintain cell conditions [19]. Experiments with human chondrocytes were performed using 3-5 different cell lots.

### Flow cytometry

Cells were incubated with 1 μg/mL human IgM or ARG098 for 30 min on ice. For investigating the effect of the neutralizing antibody, cells were incubated with 10 μg/mL of the neutralizing antibody prior to incubation with human IgM or ARG098. For individual analysis of CD4/CD8 T cells, 1 μg/mL of PerCP-labeled anti-human CD4 antibody, PE-labeled anti-human CD8 antibody or PE-labeled mouse IgG_1_, and PerCP-labeled mouse IgG_1 _were added to the reaction mixture with ARG098. After washing, the cells were incubated with FITC-labeled anti-human IgM antibody and the fluorescence intensity was determined using the fluorescence-activated cell sorter FACSCalibur 3A (Nippon BD, Tokyo, Japan).

### Cell viability assay

Twenty-four hours after the antibody treatments, a cell viability assay was performed using a formazan dye available with the Cell-Counting Kit-8. Briefly, cells were seeded in 96-well plates and incubated with antibodies for 24 h. The Cell-Counting Kit-8 reagents were then added to the culture medium. After 2 h of incubation at 37°C, absorbance was measured at 450 nm. The percentage of cell viability was calculated based on the 450 nm absorbance values of non-treated cells.

In the chondrocyte cell viability analyses, one human chondrocyte alginate bead was seeded in a well of a 96-well plate and incubated with medium containing antibody. Twenty-four hours after the antibody treatment, cell viability was assessed using the Cell Counting Kit-8.

Lymphocyte cell viability was assessed by the ATP staining method using the CellTiter-Glo™ Luminescent Cell Viability Assay Kit. Cells were seeded in 96-well plates and incubated with antibodies for 24 h. The CellTiter-Glo™ Luminescent Cell Viability Assay Kit reagents were then added and the luminescence intensity was measured after incubation for 10 min at room temperature.

### Annexin-V/PI staining

RA synoviocytes were incubated with antibodies for 6 h. Cells were harvested, and stained with PI and FITC-conjugated annexin-V [20]. Fluorescence intensity (annexin-V positive) was analyzed using the FACSCaliber 3A.

### Preincubation of RA synoviocytes with cytokines

RA synoviocytes were incubated with 5 ng/mL of TNF-α or 0.5 ng/mL of IL-1β for 5 days. The concentrations of TNF-α and IL-1β were determined based on their possible concentrations in synovial fluids of RA-affected joints [21,22].

### Animals

Four-week-old male CB17/Icr Crj-SCID mice were purchased from Charles River Japan (Yokohama, Japan). The mice were housed in pathogen-free facilities, and water and food were provided *ad libitum*. Mice aged 5-8 weeks were used for the experiments.

### Preparation of the SCID-HuRAg cartilage co-implantation mouse model

All animal experimental procedures and care were approved and monitored by the Institutional Animal Care and Use Committee of Santen Pharmaceutical Co., Ltd. The SCID-HuRAg mice were produced by the method established by Sakai *et al*. [13].

In addition, to study the effect of ARG098 on cartilage destruction in this model, RA synovial tissue samples of about 1 cm^3 ^and suitable pieces of RA cartilage were grafted subcutaneously on the backs of SCID mice that were anesthetized with ketamine and xylazine. A difference between the SCID-HuRAg mouse model used in previous studies and that used in this study is that the cartilages used in this study were derived from RA patients.

### ARG098 treatment *in vivo*

ARG098 or control human IgM antibody was directly injected into the synovial tissues 6 weeks after tissue implantation. To assess the induction of apoptosis by ARG098, implanted tissues were fixed in formalin 24 h after antibody injection. For histopathological analysis of the tissues, mice were euthanized and the implanted tissues were isolated 4 weeks after antibody injection. To assess cell numbers in the tissues, cells were counted in 4 areas of the hematoxylin and eosin (HE)-stained tissues.

### Detection of apoptosis by DNA nick end labeling and histopathological analysis

Apoptosis was detected by DNA nick end labeling using the *In situ *apoptosis detection kit. The number of positively stained cells was counted in each tissue sample. Serial sections were stained with HE for comparison with the nick end labeling results.

Other tissues were fixed with formalin, embedded in paraffin, and sectioned. The slides were stained with HE using standard methods.

### Statistical analyses

Data were analyzed using SAS software (version 8.2). The cytotoxic effects of ARG098 were analyzed by comparison with the untreated cells/group using Dunnett's or Steel's test when the data were homoscedastic or heteroscedastic, respectively. The cytotoxic effects of ARG098 on RA synoviocytes were compared with those of human IgM or ARG098 after pretreatment with the Fas-neutralizing antibody SM1/23 using Student's *t*-test. The cell number in the grafted tissue and the effects of ARG098 were analyzed by comparison with untreated or human IgM-treated tissues using Student's *t*-test when the data were homoscedastic. P values <0.05 were deemed to indicate statistical significance.

## Results

### Induction of apoptosis in RA synoviocytes

**Figure 1:**
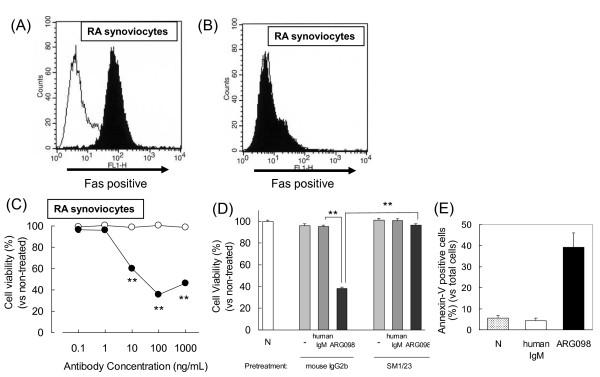
**ARG098 binding and cytotoxicity to rheumatoid arthritis (RA) synoviocytes**. (A) ARG098 binding activity to RA synoviocytes was assessed by flow cytometory. The white area is stained with human IgM and the black area is stained with ARG098. (B) RA synoviocytes pretreated with anti-Fas neutralizing antibody clone SM1/23. (C) Representative cell viability of RA synoviocytes following ARG098 treatment vs. non-treated condition as measured by the WST assay. Black circles, ARG098; white circles, human IgM. Each point represents mean ± SEM (n = 5). **: p < 0.01 vs. non-treated cells (Dunnett's test). (D) Neutralization of RA synoviocyte viability by SM1/23. The cells were treated with 100 ng/mL of human IgM or ARG098. N, non-treated group. Each bar represents mean ± SEM (n = 5). **: p < 0.01 (Student's *t*-test). (E) RA synoviocytes were treated with 100 ng/mL of human IgM or ARG098. After stained with PI/annexin-V, the annexin-V positive fluorescent intensity was measured by FACSCalibur 3A. Each bar represents mean ± S.E.M of five data from synoviocytes derived from five different patients with RA.

The binding of ARG098 was assessed by flow cytometry, and the binding ability was detected in the synoviocytes isolated from RA patients (Figure 1A). This binding was completely displaced by neutralizing antibody treatment (Figure 1B). The binding affinity was the same as that of the anti-Fas antibody clone UB2 (unpublished data). These data indicate that ARG098 recognized the Fas molecule on the RA synoviocyte cell surface. These experiments were performed using 5 different RA synoviocyte samples derived from the 5 different RA patients, and ARG098 binding and displacement was observed in all experiments. The RA synoviocytes expressed Fas at a rate of 60 ± 8.9% in the 5 different synoviocyte samples.

RA synoviocyte viability decreased after the 24-h treatment with ARG098 at a concentration of 10 ng/mL or higher (Figure 1C). The decrease in RA synoviocyte viability dramatically disappeared following pretreatment with the neutralizing antibody clone SM1/23 (Figure 1D). Moreover, ARG098 at 100 ng/mL induced annexin-V staining within 6 h of treatment (Figure 1E). Because annexin-V staining occurs in the early phase of apoptosis [20], this result indicates that ARG098 induced apoptosis in the RA synoviocytes. The results of these cell cytotoxicity experiments were similar among the synoviocyte samples obtained from the 5 different RA patients.

### Induction of apoptosis in cytokine-treated RA synoviocytes

To more clearly identify ARG098 potency in the target tissues, we attempted to mimic the condition of the RA synovium in inflammatory joints by treating the RA synoviocytes with cytokines *in vitro*.

**Figure 2:**
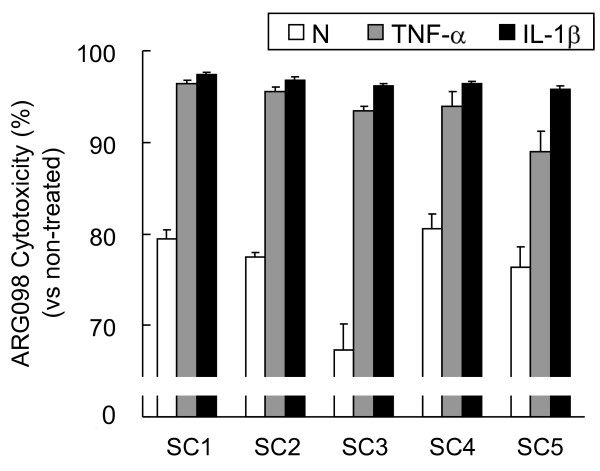
**Cytotoxic effect of ARG098 on RA synoviocytes preincubated with inflammatory cytokines**. RA synoviocytes (RASC) from five patients were preincubated with 5 ng/mL of tumor necrosis factor (TNF)-α or 0.5 ng/mL of interleukine (IL)-1β for 5 days and then treated with 100 ng/mL of ARG098 for 24 h. N, non-pretreated group. Each bar represents mean ± SEM (n = 6). Cytotoxicity was measured with the WST assay.

RA synoviocytes were preincubated with TNF-α or IL-1β for 5 days and then treated with 100 ng/mL ARG098 for 24 h. The cytotoxic effects of ARG098 were measured and compared with those under normal culture conditions. ARG098 induced cytotoxicity in 70%-80% of RA synoviocytes under normal conditions and in more than 90% of RA synoviocytes when preincubated with TNF-α or IL-1β (Figure 2). RA synoviocytes that were preincubated with cytokines were more sensitive to ARG098 than those cultured under normal conditions. These results were similar for the synoviocytes obtained from all 5 RA patients.

### Induction of apoptosis in synovium-infiltrating lymphocytes but not in PBMCs

**Figure 3:**
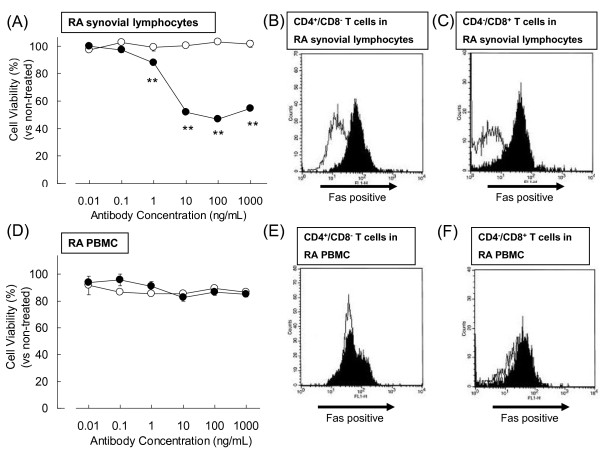
**Effect of ARG098 on infiltrating lymphocytes isolated from the synovium of RA patients and peripheral blood mononuclear cells (PBMCs) isolated from RA patients**. (A) Representative infiltrating lymphocytes viability in the RA synovium following ARG098 treatment vs. the non-treated condition. Black circles, ARG098; white circles, human IgM. Each point represents mean ± SEM (n = 5). **: p < 0.01 vs. non-treated cells (Dunnett's test). (B) ARG098 binding activity to CD4^+^CD8^- ^T cells in lymphocytes isolated from RA patients was assessed by flow cytometory. The white area is stained with human IgM, and the black area is stained with ARG098. (C) ARG098 binding activity to CD4^-^CD8^+ ^T cells in lymphocytes isolated from RA patients. (D) Representative PBMC viability from the same RA patient following ARG098 treatment vs. the non-treated condition. Black circles, ARG098; white circles, human IgM. Each point represents mean ± SEM (n = 5). (E) ARG098 binding activity to CD4^+^CD8^- ^T cells in the PBMCs from RA patients. The white area is stained with human IgM and the black area is stained with ARG098. (F) ARG098 binding activity to CD4^-^CD8^+ ^T cells in the PBMCs from RA patients.

Many lymphocytes infiltrate the RA synovium and supply inflammatory cytokines [23], and these cells are also therapeutic targets of ARG098. ARG098 induced apoptosis in RA synovium-infiltrating lymphocytes, which did not adhere and were separated from the RA synoviums after 1 day of incubation as written in methods, (Figure 3A) of 5 different RA patients. CD4^+^CD8^- ^T cells (Figure 3B) and CD4^-^CD8^+ ^T cells (Figure 3C) in RA synovium-infiltrating lymphocytes were mostly Fas positive and bound by ARG098. These results indicate that RA synovium-infiltrating lymphocytes express the active Fas molecule and that ARG098 is effective for removing these active inflammatory lymphocytes from RA joints.

Simultaneously with these experiments, the effect of ARG098 on PBMCs was tested with PBMCs isolated from the same RA patients. ARG098 did not decrease PBMCs viability (Figure 3D) and the results were similar in cells isolated from the 5 different RA patients. To compare the Fas expression in PBMCs to that of RA synovium-infiltrating lymphocytes, we investigated ARG098 binding in each PBMC population by flow cytometry (Figure 3 E and F). The population size of the CD4^+^CD8^- ^and CD4^-^CD8^+ ^T cells in the PBMCs was nearly similar to that of the RA synovium-infiltrating lymphocytes. However, ARG098 did not bind to most of these cells, which suggests that these cells were Fas negative. These observations suggest that ARG098 induces the death of RA synovium-infiltrating lymphocytes but not in the PBMCs in the same RA patient at the same time.

### ARG098 binding ability and cytotoxic influence in human chondrocytes

ARG098 is considered an anti-RA agent for intra-articular injection. With local administration, ARG098 is directly delivered, which minimizes the adverse effects because of low systemic exposure. If controlled well, the toxicity of ARG098 to normal cells can be prevented because the exposed tissues are separated by the blood circulatory system. RA chondrocytes express Fas molecules and are sensitive to apoptosis [24]. ARG098 injection could thus possibly induce apoptosis in chondrocytes and lead to the destruction of cartilage, which would decrease the QOL of patients.

**Figure 4:**
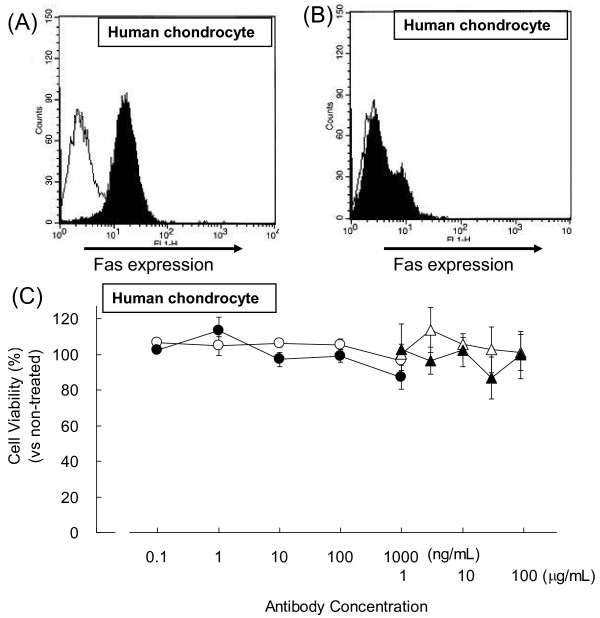
**ARG098 cytotoxicity to human chondrocytes**. (A) ARG098 binding activity to human chondrocytes was assessed by flow cytometory. The white area is stained with human IgM and the black area is stained with ARG098. (B) RA synoviocytes pretreated with anti-Fas neutralizing antibody clone SM1/23. (C) Representative cell viability of human chondrocytes following ARG098 treatment (Experiment 1: from 0.1 to 1000 ng/mL, Experiment 2: from 1 to 100 μg/mL) versus untreated condition, as measured by the WST assay. Black circles and black triangles, ARG098; white circles and white triangles, human IgM. Each point represents mean ± SEM (n = 5).

Therefore, to evaluate the safety of ARG098, we assessed its effects on chondrocytes. The binding of ARG098 to human chondrocytes was detected (Figure 4A), and that was similar to that of the anti-Fas antibody clone UB2 (unpublished data). This binding could be displaced by treatment with a Fas-neutralizing antibody (Figure 4B). Given these data, it appears that ARG098 recognizes the Fas molecules on the cell surface of human chondrocytes. However, ARG098 did not reduce cell viability in human chondrocytes at concentrations of 0.1 and 1000 ng/mL or at much higher concentrations of 1 and 90 μg/mL (Figure 4C). Thus, ARG098 bound to normal human chondrocytes but did not induce cell death. ARG098 did induce cell death in RA synoviocytes at a concentration of 10 ng/mL or higher. However, even at 90 μg/mL, ARG098 did not induce cell death. These results suggest that ARG098 delivered by an intra-articular injection is safe, as evidenced by the large difference in sensitivity between the RA synoviocytes and chondrocytes in terms of ARG098-induced cell death.

### ARG098-induced apoptosis in RA synovium implanted in SCID mice

**Figure 5:**
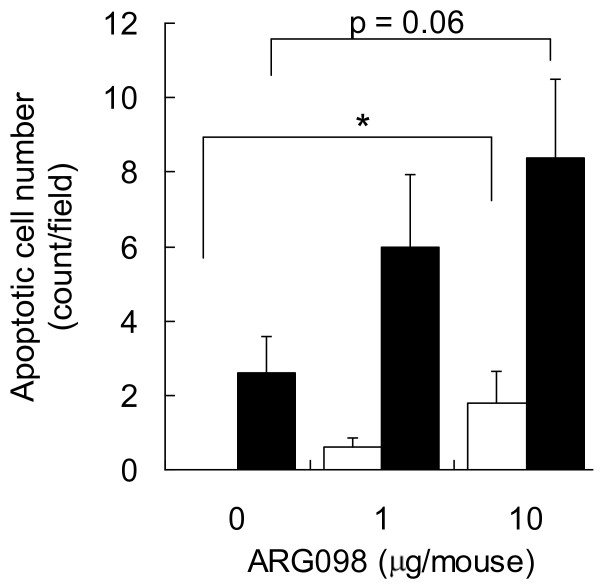
**Apoptotic cell number after ARG098 treatment in RA synovium implanted in SCID mice**. Apoptotic cell numbers were counted and are expressed as the cell number in a field. The white bars show the apoptotic cell number in the collagen tissue. The black bars show the apoptotic cell number in the lymph follicle. Statistical analyses of cell number in the lymph follicle were performed with Dunnett's test and p values are shown in the figure. Analysis of the collagen tissue was performed with Steel's test. *, p < 0.05 versus 0 μg/mouse group.

The efficacy of ARG098 *in vivo *was evaluated using the SCID-HuRAg mouse model. Twenty-four hours after ARG098 injection, apoptotic cells were detected in the infiltrating lymphocytes of the lymph follicle and synoviocytes in the collagen tissue in the RA synoviums engrafted into the SCID mice. In contrast, the control human IgM antibody did not induce apoptosis (Figure 5). The apoptotic cell number was increased in a dose-dependent manner by ARG098 treatment. These results indicate that ARG098 induces apoptosis *in vivo *similar to that *in vitro*.

### ARG098 treatment decreases the infiltrating cells and inhibits synovial growth

**Figure 6:**
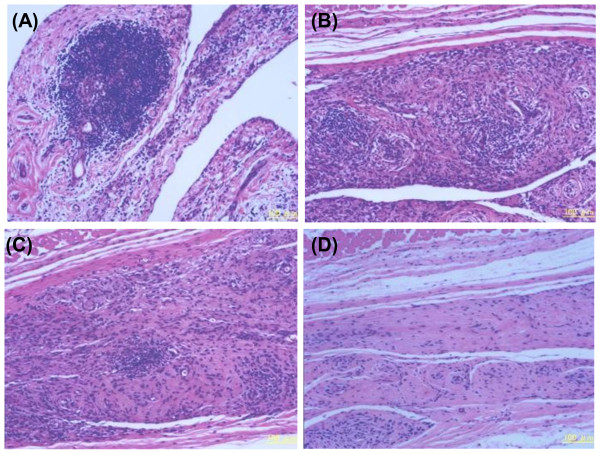
**Histopathological analyses of RA synovial grafts in the SCID-HuRAg model and the effects of ARG098**. RA synovial tissue samples were stained with hematoxylin and eosin (HE). (A) RA synovial tissue before implantation, (B) an untreated graft, (C) 10 μg human IgM-injected graft, and (D) 10 μg anti-APO-1 IgM-injected graft.

In the RA synovial tissue that was not implanted, infiltrating lymphocytes were abundant and gathered to form lymph follicles (Figure 6A). Ten weeks after implantation, infiltrating cells remained in the RA synovium. This observation is similar to what was observed for the original synovial tissue (Figure 6B). Moreover, synovial growth into collagen tissue, a typical characteristic of the RA synovium, was observed, thereby resembling synovial hyperplasia. When 10 μg of human IgM was injected into the implanted tissue (Figure 6C), many infiltrating cells, lymph follicles, and a hypertrophied synovium were observed, and these findings were similar to those for the untreated tissue after implantation (Figure 6B) or the original tissue (Figure 6A). When 10 μg of ARG098 was injected into the RA synovium engrafted into the SCID mice, the number of infiltrating lymphocytes markedly decreased and synovial growth was diminished in the collagen tissue (Figure 6D). In addition, synoviocytes were replaced by adipocytes. Cell numbers in the ARG098-treated tissue significantly decreased compared with those in the untreated and human IgM-treated tissues (unpublished data).

### ARG098 protects against cartilage destruction

**Figure 7:**
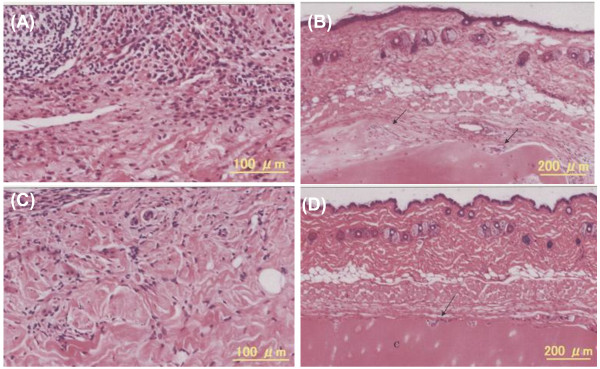
**Histopathological analyses of RA synovium and cartilage grafts in the SCID-HuRAg model and the effects of ARG098**. The RA synovium tissues and cartilage were stained with HE. RA synovium was injected with 10 μg human IgM (A, B), or 10 μg ARG098 (C, D). The arrows indicate the cartilage erosion by RA synovium. C indicates cartilage.

RA synovium implanted into the SCID mice is a good model to mimic RA-affected joints. However, to estimate the efficacy and safety in the synovium and cartilage in the joints, modification of the model was necessary. The synovium and cartilage obtained from the same RA patient were co-implanted on the backs of the SCID mice. When 10 μg of human IgM was injected into the RA synovium, infiltrating lymphocytes were abundant in the synovial tissue (Figure 7A), and the cartilage was infiltrated and digested by the synovium (Figure 7B). On the other hand, when 10 μg of ARG098 was injected into the RA synovium, the number of infiltrating lymphocytes markedly decreased (Figure 7C). This decrease is comparable to that observed in the synovium-implanted model (Figure 6D). Cartilage destruction was completely inhibited and there was little cartilage erosion due to synovium infiltration (Figure 7D). The border between the synovial tissue and cartilage could be readily detected in the ARG098-treated tissue.

## Discussion

The APO-1/Fas/CD95 (Fas) molecule is a cell membrane receptor [25], and the anti-Fas antibody or the internal Fas ligand binds to Fas to induce cell apoptosis [26]. However, IgG subtype anti-Fas antibodies may not induce apoptosis without Fas receptor multimerization [27]. IgG subtype anti-Fas antibodies usually need crosslinkers or internal factors for multimer construction and incomplete Fas multimer formation prevents apoptosis [28,29].

Then, IgM subtype anti-Fas antibody was expected to be suitable to activate the Fas signal effectively. Therefore, ARG098 was constructed as a mouse-human chimeric antibody for the purpose of developing an anti-RA agent for humans.

The cytotoxic activity of ARG098 toward the target cells, i.e., RA synoviocytes and synovium-infiltrating lymphocytes, was shown in this study. However, even after ARG098-treatment, some ARG098-resistant RA synoviocytes remained. The reasons for this resistance to Fas-mediated death signals are believed to be as follows: first, Fas-mediated apoptosis in synoviocytes is regulated at the level of recruitment of the Fas-associated death domain protein (FADD) to the death-inducing signaling complex (DISC) [30]. The reason why the RA synoviocytes preincubated with TNF-α were more sensitive to ARG098-induced apoptosis than the RA synoviocytes in normal culture conditions can then be explained, at least in part, by caspase 3 and 8 up-regulation by TNF-α [31]. Second, the conditions under which RA synoviocytes are cultured have a huge impact on their susceptibility to the Fas signal [6]. The cell cycling status or proliferating stage influences Fas-mediated apoptotic signal transduction and sometimes the RA synovium becomes resistant to Fas-mediated cell death. Third, the PI3K/Akt-1 pathway is activated in the cells of RA joints and this pathway activates apoptosis-inhibitory factors [7].

We also understand that our positive *in vitro *results with ARG098 may be a secondary event depending on the tissue culture conditions such as proliferation rate, cell cycling status and culture confluency. In our culture condition, we have not checked what cell population are there, but we speculate the majority of our tested cell population may be synovial fibroblast cells which are consisted with mixed cell status of proliferation rate and cell cycling, etc. We suppose that there are both proliferating and arresting synovial fibroblasts in our culture with 10% FBS. Although from our *in vitro *results with the experimental cell culture in 10% FBS we could not evaluate the intrinsic quantitative efficacy of ARG098 and could not extrapolate intrinsic i*n vivo *results, we believe that some part of *in vivo *effects are expected by our *in vitro *results.

While ARG098 is a new potentially effective tool for the inhibition of synovial hyperplasia and joint swelling, ARG098 could also possibly affect normal cells that express Fas on the cell surface and this may lead to adverse events during RA treatment. Then we checked the effect of ARG098 on PBMCs from the same RA patients, and on normal human chondrocytes. As results, we could not found any adverse effect of ARG098 on these cell viabilities, *in vitro*. In case of chondrocytes, in addition, no apoptosis or damage to RA chondrocytes was observed in ARG098-treated tissues during the histopathological assessment of ARG098-treated RA synovium and cartilage co-implanted in the SCID-HuRAg mice.

In addition, because functional Fas is also expressed on the surface of human hepatocytes [11] and the anti-Fas antibody induces apoptosis in hepatocytes [12], we investigated whether ARG098 could induce a severe adverse effect on normal hepatocytes. Although ARG098 bound to normal human hepatocytes, it did not induce cytotoxicity at concentrations less than 1000 ng/mL (unpublished data), indicating that the apoptosis-inducible concentration of ARG098 in RA synoviocytes or lymphocytes infiltrating the RA synovium is lower than that in hepatocytes. This sensitivity difference can be partly explained by inadequate preparation of the cellular machinery [32] or by Fas-resistant signal activation [7]. And also as ARG098 is intended to be delivered as an intra-articular injection, wherein the leak speed of ARG098 into the circulatory system is expected to be slow and thus the hepatocyte exposure concentration would be low. Thus, ARG098 appears to be safe in this regard and these expectations are also supported by other safety studies performed with ARG098 in human tissue slices or cross-reactive animals.

TUNEL-positive cells were not abundant in the ARG098-treated tissues in the SCID-HuRAg mice. Apoptotic cells can be difficult to detect because the DNA fragments detected by the TUNEL method are readily degraded. Twenty-four hours after the injection is considered to be the best time to detect apoptotic cells, but not all such cells were detected in this study. In addition, apoptotic cells are believed to be processed and removed rapidly by phagocytes. Although TUNEL-positive cells were rarely detected, ARG098 is believed to induce apoptosis in tissues after injection because ARG098 clearly induced apoptosis *in vitro*.

On the other hand, IgM can activate the complement system [33] and another part of the mechanism by which ARG098 reduces synovial hyperplasia may be explained through this system. Induction of apoptosis and activation of the complement system by ARG098 may contribute to the inhibition of synovial growth *in vivo*. Further experiments concerning apoptosis induction and complement system activation are necessary to elucidate the pharmacological efficacy of ARG098 in the treatment of synovial hyperplasia as well as on events that occur in joints following ARG098 injection.

It is recently reported that RA synovial fibroblasts spread in naïve cartilage lead to cartilage destruction [34]. This means that inhibition of the RA synovial cells by ARG098 may not only directly protect the cartilage in contact with the overgrown synovium but may also protect the naïve cartilage and inhibit the spread of the disease.

It may be expected that biological removal of the RA synovium will become a new complementary therapy with systemic anti-rheumatic drugs in future.

## Conclusions

ARG098, a novel anti-Fas/APO-1/CD95 monoclonal antibody, exhibits potency for treating RA synovial hyperplasia, which plays crucial roles in joint destruction in RA patients, by decreasing the numbers of RA synoviocytes and synovium-infiltrating lymphocytes via Fas-mediated apoptosis. Taken together, these results suggest that ARG098 might be a potential RA therapy that directly suppresses synovial activity and decreases cartilage destruction when delivered by intra-articular injection. ARG098 P1/2 clinical studies in RA patients are currently being performed in Japan and Europe. The results of these studies are eagerly anticipated.

## Competing interests

NOK, MTI, OK, MS, and HA work for Santen Pharmaceutical Co., Ltd. HN and KN work for Institute of Medical Science, St. Marianna University School of Medicine. KF and TM work for Matsubara Mayflower Hospital.

All studies were performed with the financial resources of Santen Pharmaceutical Co., Ltd.

## Authors' contributions

NOK designed the study, carried out and participated in all studies and drafted the manuscript. MTI carried out and participated in most of studies and study design. OK carried out the histopathological assays. HN, KN, KF, and TM participate in the material preparation. MS and HA conceived of the study, and participated in its design and coordination and helped to prepare manuscript. All authors read and approved the final manuscript.

## Pre-publication history

The pre-publication history for this paper can be accessed here:

http://www.biomedcentral.com/1471-2474/11/221/prepub
